# Understanding the Effects of High Pressure on Bacterial Spores Using Synchrotron Infrared Spectroscopy

**DOI:** 10.3389/fmicb.2019.03122

**Published:** 2020-01-31

**Authors:** Chloé Modugno, Caroline Peltier, Hélène Simonin, Laurence Dujourdy, Francesco Capitani, Christophe Sandt, Jean-Marie Perrier-Cornet

**Affiliations:** ^1^AgroSup Dijon, PAM UMR A 02.102, Université Bourgogne Franche-Comté, Dijon, France; ^2^AgroSup Dijon, Service d’Appui à la Recherche, Dijon, France; ^3^Synchrotron SOLEIL, Saint-Aubin, France

**Keywords:** *Bacillus subtilis*, nisin, germination, activation, mild treatments

## Abstract

Bacterial spores are extremely resistant life-forms that play an important role in food spoilage and foodborne disease. The return of spores to a vegetative cell state is a three-step process, these being activation, germination, and emergence. High-pressure (HP) processing is known to induce germination in part of the spore population and even to inactivate a high number of *Bacillus* spores when combined with other mild treatments such as the addition of nisin. The aim of the present work was to investigate the mechanisms involved in the sensitization of spores to nisin following HP treatment at ambient temperature or with moderate heating leading to a heterogeneous spore response. *Bacillus subtilis* spores were subjected to HP treatment at 500 MPa at 20 and 50°C. The physiological state of different subpopulations was characterized. Then Fourier transform infrared (FTIR) microspectroscopy coupled to a synchrotron infrared source was used to explore the heterogeneity of the biochemical signatures of the spores after the same HP treatments. Our results confirm that HP at 50°C induces the germination of a large proportion of the spore population. HP treatment at 20°C generated a subpopulation of ungerminated spores reversibly sensitized to the presence of nisin in their growth medium. Regarding infrared spectra of individual spores, spores treated by HP at 50°C and germinated spores had similar spectral signatures involving the same structural properties. However, after HP was performed at 20°C, two groups of spores were distinguished; one of these groups was clearly identified as germinated spores. The second group displayed a unique spectral signature, with shifts in the spectral bands corresponding to changes in membrane fluidity. Besides, spores spectra in the amide region could be divided into several groups close to spectral properties of dormant, germinated, or inactivated spores. The part of the spectra corresponding to α-helix and β-sheet-structures contribute mainly to the spectral variation between spores treated by HP at 20°C and other populations. These changes in the lipid and amide regions could be the signature of reversible changes linked to spore activation.

## Introduction

High pressure (HP) is the most widely used non-thermal method of processing to stabilize commercial foods. By reducing the microbial load, this process extends the product shelf-life while meeting consumer demand for freshly prepared foods with high sensory and nutritional qualities (Huang et al., 2017). Industrial HP machines are currently designed to treat foods at a pressure of up to 700 MPa at ambient or refrigerated temperatures, thus ensuring complete inactivation of foodborne pathogens, spoilage microorganisms, and deteriorative enzymes (Metrick et al., 1989; Patterson et al., 1995). However, the persistence of bacterial spores is the Achilles heel of this process because spores of *Clostridium botulinum*, *Bacillus cereus*, and other pathogenic and alteration species easily resist HP treatments and can then readily return to active growth (Nguyen Thi Minh et al., 2010).

When subjected to an HP process with moderate heating at about 50°C, two different processes induce the germination of *Bacillus* sp. bacterial spores depending on the pressure applied. Moderate HP (<400 MPa) induces spore germination by triggering the germinant receptors (GRs) located in the inner spore membrane, whereas HP above 500 MPa induces GR-independent germination by triggering the release of dipicolinic acid (DPA) from the spore core (Paidhungat et al., 2002; Doona et al., 2014; Setlow, 2014). Spores lose their inherent resistance once germinated and can then be inactivated by other inactivation processes (Black et al., 2005; Nguyen Thi Minh et al., 2010). HP has been shown to act synergistically with nisin to inactivate various species of spore-forming bacteria (Black et al., 2008; Gao et al., 2011; Aouadhi et al., 2013). Furthermore, the generally accepted hypothesis is that HP-germinated spores are sensitive to nisin and thus inactivated by the latter. This has not, however, been clearly demonstrated. Indeed, spore subpopulations respond differently to mild stress such as HP. Some spores may begin the germination process while others remain dormant, rendering the exact mechanism of the synergy difficult to evidence.

The aim of the present work was to investigate the mechanisms involved in the sensitization of spores to further stresses such as the addition of nisin following HP treatment at ambient temperature or with moderate heating leading to a heterogeneous spore response. For this purpose, we characterized the physiological state of *Bacillus subtilis* spore subpopulations after HP treatment at 500 MPa at 20 and 50°C. Then we used Fourier transform infrared (FTIR) microspectroscopy coupled to a synchrotron light as a source of infrared (IR) photons to explore the heterogeneity of the biochemical signatures of the spores after the same HP treatments. FTIR spectroscopy is a powerful analytical method to characterize cell metabolism and biochemical properties of biomolecules such as lipids, proteins, and nucleic acids. Some authors have already used this method with spores, which has provided new knowledge about species-related biochemical compositions and the effect of HP processes on the structural properties of spores (Valentine et al., 2006; Georget et al., 2014). However, these studies were conducted on bulk samples, providing a single average spectrum characterizing millions of spores. In this study, we used synchrotron attenuated total reflectance (ATR)–FTIR microspectroscopy to measure the spectra of individual spores and assess the heterogeneity of their response to the HP treatment.

## Materials and Methods

### Bacterial Strains, Growth, and Sporulation Conditions

*Bacillus subtilis* PS533 and the mutant strain *B. subtilis* PS4150 (provided by the Department of Molecular, Microbial, and Structural Biology, University of Connecticut Health Center, United States) were used in this study. Strain PS4150 has a defective spore coat due to the deletion of most of the *cotE* and *gerE* coding sequences (Ghosh et al., 2008).

For sporulation, cultures were obtained by inoculating two or three fresh colonies in 50 ml of complex medium (CM): 10 g/L of meat extract (Biokar, France), 2 g/L of yeast extract (Biokar, France), and 0.04 g/L of MnSO_4_ (Sigma-Aldrich, France) (André et al., 2013). After incubation for about 24 h (until 0.4 < OD_600_ < 0.6), 0.4 ml of the culture was spread on CM agar (CMA) in Petri dishes (CM supplemented with 15 g/L of agar, Biokar, France). Sporulation was monitored using phase contrast microscopy. When more than 95% of phase-bright spores were observed, spores were harvested by flooding the agar plates with cold sterile distilled water and scraping the agar surface with a sterile cell spreader. Spore suspensions were then centrifuged (3,600 *g*, 15 min, 4°C), and the spores were suspended in 0.2 μm of filtrated 70% ethanol (filter: cellulose acetate, Sartorius, France; ethanol: Elvetec, France) for 1 h to inactivate vegetative cells (Koransky et al., 1978). Spores were then washed three times by successive centrifugation (3,600 *g*, 15 min, 4°C) and suspension in sterile distilled water at 4°C. Spores were enumerated [in colony-forming unit (CFU)/ml] on Luria broth (LB) medium (Sigma-Aldrich, France). Spore suspensions were standardized to obtain a final concentration of 10^8^ CFU/ml and stored at 4°C. For the synchrotron FTIR microspectroscopy experiments, the final spore suspensions were standardized to obtain a final concentration of 10^9^ CFU/ml, frozen overnight at −80°C, and freeze-dried (FreeZone 18, Labconco, Kansas City, United States). Spores were stored at 4°C for a maximum of 1 month until use.

### HP Treatment and Control Samples

High-pressure tests were performed in a Top Industrie 700-MPa vessel (France) with a 7-cm^3^ metal pressure chamber (working temperature: −120°C/120°C). Water was used as the pressure-transmission fluid. The pressure chamber was immersed in a water bath (Minisat 240, Huber, Germany) to control the internal temperature and to ensure that adiabatic heating during compression did not increase the temperature by more than 5°C above the desired set point. The compression rate was set to 3 MPa/s, and decompression was almost instantaneous (<3 s).

### Effect of HP on Inactivation, Germination, and Nisin Sensitivity of *B. subtilis* Spores

The initial spore suspensions were diluted (1:100) in 0.11 mol/L of 2-(*N*-morpholino)ethane sulfonic acid (MES) buffer at pH 6.1 (Sigma-Aldrich, France) to reach a final concentration of approximately 10^7^ CFU/ml. MES buffer was chosen because its pH varies only slightly with temperature and pressure (Δp*K*a/°C = −0.011; pH varies from 5.5 to 6.5 between 10 and 1,000 MPa at 25°C) (Bruins et al., 2007). Then, 0.5 ml of each spore suspension in MES was heat-sealed in polyethylene pouches (polyethylene transfer pipette, Dominique Dutscher). For each strain, zero-time samples were taken to determine the initial spore concentration before treatment. The pouches were placed in the HP vessel 5 min before treatment to allow the temperature to equilibrate. Samples were treated at 500 MPa for 10 min at 20°C or 50°C and then immediately immersed in iced water.

Immediately after treatment, the samples were divided into three equal volumes. The first volume was serially diluted and plated on an LB medium (15 g/L, Sigma-Aldrich, France; bacteriological agar type E, 15 g/L, Biokar, Beauvais, France) to determine bacterial cell numbers in colony-forming units per milliliter. The second volume was treated at 80°C for 10 min in a water bath to inactivate germinated spores and then serially diluted and plated on LB Petri dishes for enumeration. The fraction of HP-induced germinated spores was calculated as the difference between the logarithmic counts of pressure- and heat-treated (*N*_HPT_) and pressure-treated (*N*_HP_) portions of the samples [log10(*N*_HPT_) - log10(*N*_HP_)].

The third volume was plated on an LB medium supplemented with 50 IU/ml of nisin (Sigma-Aldrich, France) to assess the sensitization of spores to nisin. To evaluate the effect of nisin alone, control samples of non-HP-treated spores were plated on an LB medium supplemented with nisin. Furthermore, to assess the reversibility of nisin sensitization after HP treatment, spore suspensions were stored for 2, 4, 6, or 24 h after HP treatment before being plated on an LB medium supplemented with nisin for enumeration (in CFU/ml). As controls, HP-treated spores were stored at 4°C for 2, 4, or 6 h and plated on an LB medium without nisin.

### Synchrotron FTIR Microspectroscopy of Bacterial Spores

#### Sample Preparation

The lyophilized spore stock was suspended in MES buffer to obtain a final concentration of approximately 10^7^ CFU/ml and treated at 500 MPa for 10 min at 20°C or 50°C, as explained above. After treatment, the samples were centrifuged at 11,200 *g* for 5 min at 4°C, washed twice in deionized water to remove the MES buffer, and suspended in distilled water.

As a control, germinated spores were obtained by suspending lyophilized spores in an LB medium (15 g/l, Sigma-Aldrich, France) at a final concentration of 10^7^ CFU/ml. Spore samples in LB were incubated for 45 min at 37°C and then washed twice by successive centrifugation (11,200 *g*, 5 min, 4°C) and suspension in phosphate buffer saline (PBS).

To obtain inactivated spores, lyophilized spores were suspended in sterile distilled water at a final concentration of 10^7^ CFU/ml and treated in a dry bath (BSH1001-E, Benchmark Scientific, Edison, NJ, United States) at 121°C for 30 min. Inactivated spore samples were then centrifuged (11,200 *g*, 5 min, 4°C) and suspended in sterile distilled water.

All the samples were stored at 4°C for a maximum of 30 min until analysis.

#### Sample Analysis Using Synchrotron FTIR Microspectroscopy

Synchrotron IR microscopy was performed on the SMIS beamline at the SOLEIL Synchrotron Facility (session 20160780, Saint Aubin, France), as previously described by Passot et al. (2015). Briefly, spectra were recorded on a continuum microscope (Thermo Fisher Scientific, Villebon-sur-Yvette, France) coupled to a Nicolet 5700 FTIR spectrometer (Thermo Fisher Scientific). The microscope includes a motorized sample stage and a liquid nitrogen-cooled mercury cadmium telluride (MCT-A) detector. It operates in confocal mode with a dual-path single aperture and uses an x32 infinity-corrected Schwarzschild-type objective and a matching x32 condenser. Individual spectra were obtained at a spectral resolution of 4 cm^–1^, co-adding 256 scans over the mid-IR spectral range from 4,000 to 400 cm^–1^.

Two microliters of the HP-treated spore suspension was deposited on the base of an IR transparent ZnSe ATR crystal with a diameter of 4 mm hemispherical (refractive index: *n* = 2.4; ISP Optics Corp., Latvia) (Passot et al., 2015). The sample drop was dried at room temperature for 5 min under a gentle airflow. The hemisphere was then placed under the microscope, its base on a glass slide. Single bacterial spores were located using the visible objective. For each sample treated by HP, 50 spores were selected, and their IR spectra were recorded. For control samples, 25 spectra were recorded. Moreover, three spore-free zones were located and selected for the background IR signal.

The ATR mode was chosen to improve the spatial resolution to focus on single spores. Synchrotron light was shone through a 10 × 10-μm^2^ aperture on the ZnSe ATR crystal, resulting in beam spot of about 4.2 × 4.2 μm^2^ at the surface of the hemisphere due to the high refractive index of the crystal, allowing single spores to be measured. This high resolution was obtained without compromising the signal-to-noise ratio of the IR spectra thanks to the brightness of the synchrotron IR radiation (Passot et al., 2015).

### Chemometric Tools

#### Preprocessing

Mathematical pretreatments are necessary to eliminate experimental bias in spectral data. Classical preprocessing techniques include baseline correction, normalization, derivatives, and smoothing (Beebe et al., 1998; Wehrens, 2011).

A combination of these techniques was computed as follows: baseline correction (to reduce systematic variation from background signal), Savitzky–Golay (SG) filters (to reduce the amount of random variation such as noise), and normalization (to remove systematic variation associated with the size of the individual spores). With SG pretreatment, each point is replaced by a smoothed estimate obtained from a local polynomial regression. This pretreatment can be applied to the raw data, as well as to derivative spectra (first or second). The choice of two parameters is required: *w* corresponding to the window size in polynomial smoothers and *p* the polynomial order. In this study, the optimal window size and polynomial order were *w* = 13 and *p* = 3, respectively.

All the analyses were computed using R 3.3.2 (R Core Team and R Development Core Team, 2016). The “prospectr” package (Stevens, 2015) was used for the SG filters, and the “hyperSpec” package (Beleites and Sergo, 2015) was used for the other pretreatments.

#### Data Reduction

A single FTIR spectrum is represented by 1,867 variables (IR region from 650 to 4,000 cm^–1^). The two most informative regions were from 2,800 to 3,050 cm^–1^ and from 1,500 to 1,800 cm^–1^, which correspond to the lipid and the protein domains, respectively. To retain only useful information, these regions were pretreated separately.

#### Principal Component Analysis

Principal component analysis (PCA) is a widely used exploratory chemometric tool. It reduces the number of variables while projecting the data in a new space that explains the variance in the data set. The new axes, called principal components (PCs), are obtained from linear combinations of the original variables, and the variation along the first PCs is maximal. Here, the variables are the different wavelengths. They are consequently numerous and can be ordered. In contrast with traditional PCA – where loadings can be represented by a correlation circle – the loadings here are represented by a curve presenting the value of the PC loading (ordinate) according to the wavelength (abscissa). Thus, each PC is related to a curve that can be interpreted as a spectrum (Cordella, 2012). The positive and negative peaks observed on these spectra correspond to the spectral domains involved in the discrimination of the spectra. PCA was performed using R 3.3.2 (prcomp function in the stats package). The best data separation was obtained using baseline correction followed by SG (*w* = 13, *p* = 3), second derivative, and normalization.

Principal component analysis was performed on the spectra of spores treated by HP at 50°C or 20°C and on inactivated, dormant, and germinated spores (control samples). The lipid (3,050–2,800 cm^–1^) and the amide regions (1,800–15,00 cm^–1^) were processed. Some of the bands correspond to well-known IR absorption peaks associated with the spectral features of the spore clusters considered. These IR absorption peaks identified as indicators of specific protein and lipid structures are summarized in Table 1.

**TABLE 1:** Main spectral regions indicating specific protein and lipid spore structures.

Domain	Spectral band (cm^–1^)	Assignment	References
Lipid	2,965–2,960	CH_3_ asymmetric stretching band	Movasaghi et al., 2008; Georget et al., 2014
	2,920	CH_2_ asymmetric stretching band	Wolkers and Hoekstra, 1995; Laroche et al., 2005; Fang et al., 2007
	2,855–2,852	CH_2_ symmetric stretching band	Wolkers and Hoekstra, 1995; Laroche et al., 2005; Fang et al., 2007
Amide	1,665–1,660	α-Helix	Cheung et al., 1999; Movasaghi et al., 2008; Georget et al., 2014

## Results

### Effect of HP on *B. subtilis* PS533 Spore Inactivation–Germination and Sensitivity to Nisin

**FIGURE 1:**
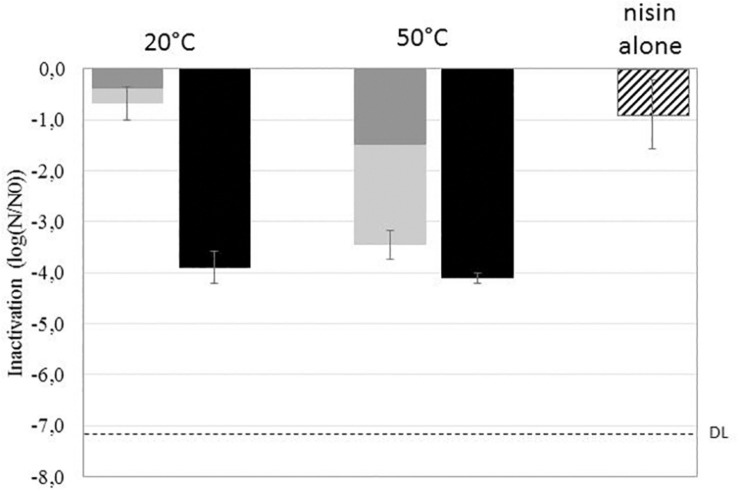
Effect of high pressure (HP) (500 MPa, 10 min, 20°C or 50°C) on the inactivation (dark gray), germination (light gray), and sensitization to nisin (black) of *Bacillus subtilis* PS533 spores. The striped bar represents the effect of nisin alone on spore inactivation. DL, detection limit.

The inactivation, germination, and sensitivity to nisin of *B. subtilis* spores were evaluated after HP treatment at 500 MPa for 10 min at 20°C or 50°C. Figure 1 presents the HP-inactivated spore fraction (dark gray bars), the HP-germinated spore fraction (light gray bars), and total spore inhibition resulting from the addition of nisin to the plating medium (black bars). The cumulated inactivation (dark and light gray bars) represents the inactivation of germinated spores achieved after HP treatment followed immediately by heat treatment at 80°C. The effect of nisin alone on spores (striped bars) represents the inhibition of spores plated only on the medium supplemented with nisin (50 IU/ml).

The effect of HP alone on spores is strongly related to the treatment temperature. HP treatment at 20°C resulted in less than 1-log inactivation and germination of spores, whereas HP treatment at 50°C resulted in 1.5-log inactivation and 3.5-log cumulated inactivation and germination of spores.

The addition of nisin to the plating medium of untreated spores induced a 1-log reduction in *B. subtilis* spore growth. HP treatment (whether at 50°C or 20°C) followed by plating on nisin-supplemented medium induced a 4-log reduction in spore outgrowth.

### Effect of Storage Time After HP Treatment on the Sensitivity of *B. subtilis* PS533 Spores to Nisin

**FIGURE 2:**
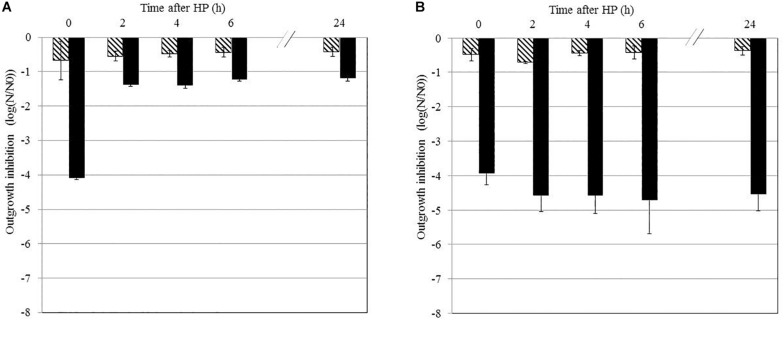
Effect of the storage time after high-pressure (HP) treatment on the sensitivity of *Bacillus subtilis* PS533 spores to nisin. Reduction of HP-treated spores after storage at 4°C for 0, 2, 4, 6, or 24 h measured by plating on Luria broth (LB) medium (striped bars) or LB medium supplemented with 50 IU/ml of nisin (black bars). **(A)** HP processed at 20°C; **(B)** HP processed at 50°C. Error bars represent SD calculated from triplicates.

*Bacillus subtilis* PS533 spores suspended in MES were treated by HP at 500 MPa for 10 min at 50°C or 20°C; stored at 4°C for 0, 2, 4, 6, or 24 h; and plated on LB medium supplemented or not with 50 IU/ml of nisin. The results are shown in Figure 2. Storage at 4°C did not impact *B. subtilis* spore growth after HP at 20°C (Figure 2A) and 50°C (Figure 2B) when plated on an LB medium without nisin.

Plating on an LB medium supplemented with nisin immediately after HP at 50°C and 20°C (storage for 0 h) induced a 4-log reduction in *B. subtilis* spore outgrowth. Spores treated by HP at 50°C remained sensitive to nisin for up to 24 h after treatment, as the inactivation remained stable (approximately 4 log). However, this sensitization seems reversible after HP treatment at 20°C, as inactivation decreased to 1 log after 2 h of storage.

### Influence of the Coat in the Sensitivity of *B. subtilis* Spores to Nisin

**FIGURE 3:**
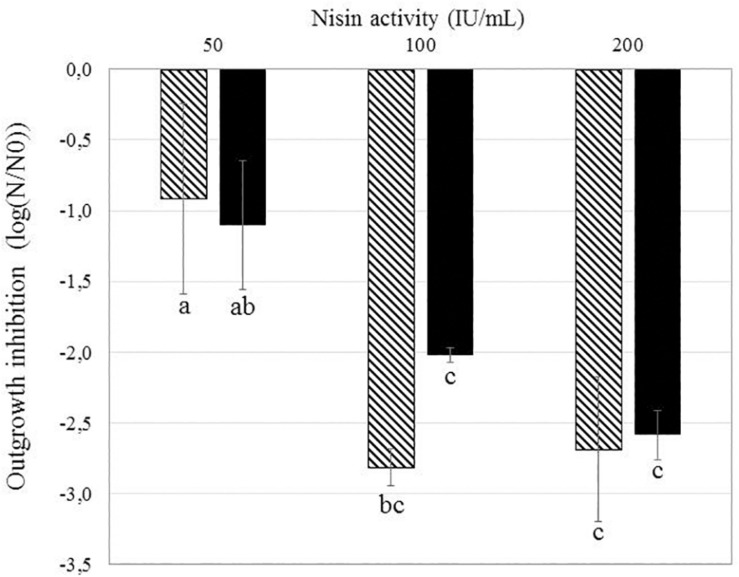
Effect of different nisin concentrations on the reduction of *Bacillus subtilis* PS533 and *B. subtilis* PS4150 (Δ*cotE*; Δ*gerE*) spore outgrowth. Striped bars: inhibition of *B. subtilis* PS533 spore outgrowth. Black bars: inhibition of *B. subtilis* PS4150 (Δ*cotE*; Δ*gerE*) spore outgrowth. Error bars represent SD calculated from independent triplicates. The letters represent a significant difference (*p* < 0.05) obtained with Tukey’s honest significant difference (HSD) test.

*Bacillus subtilis* PS533 and *B. subtilis* PS4150 (Δ*cotE*; Δ*gerE*) were plated on LB Petri dishes supplemented with nisin concentrations varying from 50 to 200 IU/ml. The effect of nisin concentrations on growth inhibition is presented in Figure 3.

Non-significant differences in growth inhibition between the two strains were detectable at the three concentrations tested, which would suggest that the spore coat is not a key barrier to preventing nisin access to the inner membrane (IM).

### Analysis of the Lipid Domain by FTIR Microscopy

**FIGURE 4:**
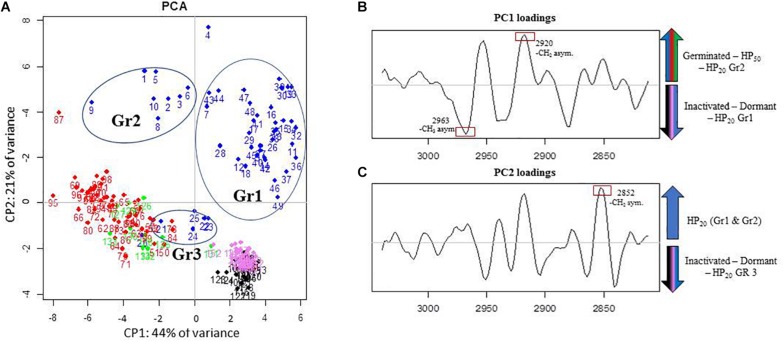
Principal component analysis (PCA) of infrared spectra obtained on control spore samples (germinated: green dots; dead: black dots; dormant: pink dots) and spores treated by high pressure (HP) at 500 MPa for 10 min at 50°C (red dots) or 20°C (blue dots) in the spectral region of lipids (3,050–2,800 cm^–1^). **(A)** Principal component 1 (PC1) vs. principal component 2 (PC2) score plots in the spectral regions of lipids (3,050–2,800 cm^–1^); **(B)** PC1 loadings plot; **(C)** PC2 loadings plot.

The PCA of the lipid region is presented in Figure 4A. First, regardless of the control samples, the PCA score plot shows clear cluster separation between the spores treated by HP at 50°C (HP_50_, red dots) and those treated at 20°C (HP_20_, blue dots). The HP_50_ and germinated spore clusters (green dots) are clearly overlapping, showing high similarity between these two groups. Conversely, three distinctive HP_20_ spore groups with unique, heterogeneous properties can be observed: Gr1, Gr2, and Gr3. One part of the population (Gr3) is close to the germinated spores, and another part (Gr1) has some similarities with dormant spores (pink dots).

**FIGURE 5:**
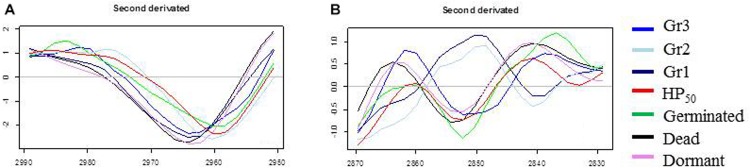
Second derivative of the mean infrared spectra of control spore samples (germinated; dead; dormant) and spores treated by high pressure (HP) at 500 MPa for 10 min at 50°C or 20°C. **(A)** Second derivative spectra in the spectral domain 2,950–2,990 cm^–1^; **(B)** Second derivative spectra in the spectral domain 2,870–2,830 cm^–1^.

The PC1 loadings plot (Figure 4B) distinguishes HP_20_-Gr2, HP_50_, and germinated spore clusters from HP_20_-Gr1, inactivated, and dormant spores. The C–H asymmetric stretching bands of CH_3_ (2,963 cm^–1^) and CH_2_ (2,920 cm^–1^) contributed significantly to the spectral variation between these groups. Regarding the second derivative spectra of the CH_3_ stretching band (Figure 5A), a marked downshift of 3 cm^–1^ was observed for the spectra of HP_20_-Gr2, HP_50_, and germinated spores.

The PC2 loadings plot (Figure 4C) distinguishes Gr1 and Gr2 from the other samples and reveals that Gr1 and Gr2 are both correlated to the C–H symmetric stretching band of the CH_2_ alkyl group (2,852 cm^–1^) (Movasaghi et al., 2008). The second derivative of the mean IR spectra of the CH_2_ alkyl group spectral bands contributing to the spectral variation observed between HP_20_-Gr1 and HP_20_-Gr2 and the other groups is presented in Figure 5. A negative peak at 2,850 cm^–1^ is visible on the spectra of all the groups except for HP_20_-Gr1 and HP_20_-Gr2 for which positive and negative peaks are present at 2,852 and 2,840 cm^–1^, respectively. This derivative shape around 2,850 cm^–1^ is the typical signature of a peak shift from above 2,850 to below 2,850 cm^–1^ as captured by PCA.

The ambiguous position of the Gr3 cluster, relatively close to the origins of both the PC1 and PC2 score plots, provides little information about its spectral characteristics. Nonetheless, the PC2 loadings plot indicates that Gr3 spectra displayed spectral contributions similar to those of germinated and HP_50_ spore clusters.

### Analysis of the Amide Domain Using FTIR Microscopy

**FIGURE 6:**
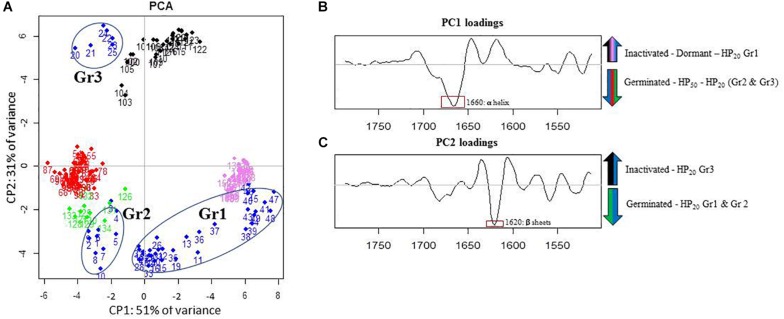
Principal component analysis (PCA) of infrared spectra obtained on control spore samples (germinated: green dots; dead: black dots; dormant: pink dots) and spores treated by high pressure (HP) at 500 MPa for 10 min at 50°C (red dots) or 20°C (blue dots) in the amide I and II spectral regions (1,800–1,500 cm^–1^). **(A)** Principal component 1 (PC1) vs. principal component 2 (PC2) score plots in the amide I and II spectral regions (1,800–1,500 cm^–1^); **(B)** loading plot of PC1; **(C)** loading plot of PC2.

The three groups of HP_20_ spores identified in the lipid domain were found again for the PCA performed in the amide domain (1,800–1,500 cm^–1^) presented in Figure 6. Regarding the PC1 and PC2 loadings plots (Figures 6B,C), the bands corresponding to α-helix (1,660 cm^–1^) and β-sheet (1,620 cm^–1^) structures contributed significantly to the spectral variation between germinated, HP_50_, and HP_20_-Gr2 spores and the other groups.

The analysis of Figures 6A,B indicates high similarities between the amide properties of the HP_50_, HP_20_-Gr2, and germinated spores. Conversely, higher cell dispersion was observed within the HP_20_-Gr1 group: one part displayed features similar to those of dormant spores, and the other one features similar to those of germinated spores. According to the PC2 loadings plot, spores in group 3 (Gr3) seem to display amide properties similar to those of dead spores.

**FIGURE 7:**
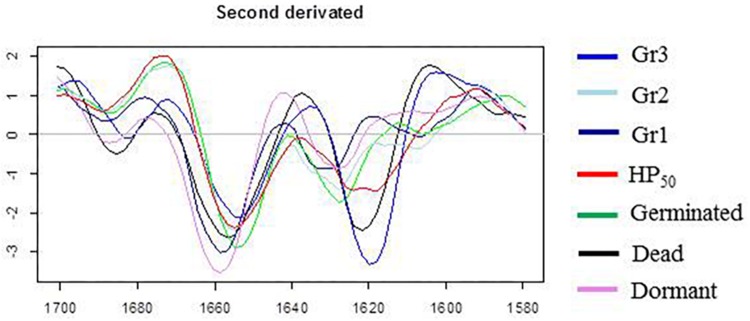
Second derivative of the mean infrared spectra (1,700–1,580 cm^–1^) of control spore samples (germinated; dead; dormant) and spores treated by high pressure (HP) at 500 MPa for 10 min at 50°C or 20°C.

The amide domains of the second derivative spectra of the control samples (germinated, inactivated, dormant spores) and the three HP_20_ groups are presented in Figure 7. While the band corresponding to α-helix is centered around 1,654 cm^–1^ in spectra of germinated, HP_50_, and HP_20_-Gr2 and HP_20_-Gr3 spores, a shift toward 1,660 cm^–1^ is observed in the spectra of dormant and HP_20_-Gr1 spores. The band located at 1,620 cm^–1^ also shows that the spectra of Gr3 and inactivated spores display similar features, as a shift of the β-sheet contribution toward 1,620–1,630 cm^–1^ is observed for these two populations.

## Discussion

High-pressure is known to induce *Bacillus* spore germination. It is thus thought that the synergy between HP and other mild treatments, such as the presence of nisin following HP-induced germination, results in the inactivation of a greater number of spores (Black et al., 2008; Gao et al., 2011; Aouadhi et al., 2013). In the case of nisin, it is generally accepted that it can only have a bactericidal effect on germinated spores (Gut et al., 2008; Egan et al., 2016), thus reinforcing the aforementioned assumption. Nisin prevents spores from becoming metabolically active by disrupting the establishment of their membrane potential (Gut et al., 2008). Nisin binds to lipid II in both spores and vegetative cells. However, this is insufficient to induce membrane pore formation in spores and thus to inhibit growth recovery (Gut et al., 2011). The formation of pores could also be due to nisin binding to the thiol groups of inner proteins that only become accessible at the time of spore germination (Morris et al., 1984).

The present study first investigated the link between the occurrence of HP-induced germination of *B. subtilis* spores and their sensitization to nisin. *Bacillus* spore germination occurs in two phases. In the first stage of germination, spores release DPA and become sensitive to heat. In the second stage, the external layers are degraded, allowing the rapid swelling of spores and their subsequent sensitization to other stresses (Nguyen Thi Minh et al., 2008; Setlow, 2014). After HP treatment, spores in the first stage of germination were evaluated by heat treatment at 80°C for 10 min. This allowed us to distinguish two subpopulations after HP treatment: HP-inactivated spores and spores that were at least in the first stage of germination. The results presented in Figure 1 indicate that a large part of the spore population was germinated after HP at 50°C. The number of spores inactivated or germinated by HP at 50°C was similar to the number of spores inhibited by the nisin present in the recovery medium. We can thus assume that HP-germinated spores are sensitized to nisin in this case. The fact that the sensitization to nisin was irreversible for at least 24 h after HP treatment at 50°C (Figure 2) reinforces this hypothesis. However, after HP treatment at 20°C, a large fraction of *B. subtilis* spores was not in the first stage of germination before subsequent inhibition by nisin. Thus, the inhibition of spore growth by nisin after HP at 20°C is not only due to spore germination, as one subpopulation was sensitized to nisin but not germinated. Additionally, the results show this sensitization to nisin was largely reversible after HP treatment at 20°C (Figure 2). HP acts on non-covalent binding of proteins, leading to the reversible or irreversible modification of secondary to quaternary protein structures depending on the treatment parameters. Moreover, the effect of HP on proteins strongly depends on the treatment temperature, as the HP–temperature stability diagram of proteins is generally elliptical (Heremans and Smeller, 1998). Hence, our results regarding the reversibility of nisin sensitization are consistent with a mechanism involving protein denaturation.

The subpopulation of ungerminated spores sensitized to nisin could have been modified by HP in a way that promotes access of nisin to the IM. Rao et al. (2016) found similar results on *B. subtilis* spores treated by HP CO_2_ (HPCD) combined with nisin. The authors suggest that HPCD promotes the penetration of nisin into the spores by damaging their coat and cortex. This hypothesis means that nisin is unable to act on dormant spores, as the outer layers (coat and cortex) block its access to the IM. Currently, little is known regarding how nisin gets to its sites of action. The spore coat is a self-assembled proteinaceous structure consisting of at least 70 proteins. It constitutes an initial barrier to large molecules, such as peptidoglycan lytic enzymes, which would otherwise have access to the spore cortex (Leggett et al., 2012). The spore coat has also been identified as playing a critical role in resistance to many chemicals, especially oxidizing agents such as nitrogen peroxide, ozone, chlorine, and hypochlorite. In contrast, smaller molecules, such as spore germination, must presumably pass through this barrier. Nisin is a cationic peptide with a molecular weight of 3,300 Da, and its potential interaction with the spore coat is unknown. Our results showed that the spore coat may not be a key barrier in preventing nisin access to the IM of spores, as the mutant *B. subtilis* strain with a reduced coat is as sensitive to nisin as the wild strain (Figure 4). We therefore assumed that potential damage to the spore coat could not explain the inhibition of spore outgrowth following the use of nisin after HP at 20°C.

To identify the biochemical modifications of spores treated by HP at spore level, spores were analyzed using IR microspectroscopy immediately after HP treatment (500 MPa) at 50°C or 20°C. The biochemical modifications could be accurately identified by tracking individual spores using this technique. Instead of observing a whole spore population using “traditional” FTIR spectroscopy methods, the structural properties of single spores could be observed, and PCA analyses identified several spore subpopulations with different biochemical signatures.

As expected, the changes induced by HP at 50°C were linked to the induction of spore germination. Indeed, PCA could not discriminate the HP_50_ group from the germinated control group in either the amide or lipid domain. The similarities between these two populations were also confirmed by the shift in the α-helix spectral band (1,665 cm^–1^), previously identified as a marker of spore germination (Cheung et al., 1999). Therefore, HP treatment at 50°C induced rapid spore germination with a fast impact on the membrane and protein structure and characteristics. These results are consistent with other studies showing a rapid induction of spore germination when *Bacillus* spores are subjected to HP treatment at temperatures over 40°C (Nguyen Thi Minh et al., 2010; Georget et al., 2014).

Concerning spores treated by HP at 20°C, heterogeneous spectral characteristics were observed in both the lipid and amide regions. Three spore populations, Gr1, Gr2, and Gr3, were discriminated in the lipid region, mainly due to a shift in the vibration of the CH_3_ groups for Gr2 spores and the CH_2_ methylene groups toward higher wavenumbers for Gr1 and Gr2 spores compared to dormant spores. The shift in the vibration of the CH_3_ and CH_2_ groups is commonly linked to modifications in the packing of lipid molecules in biological membranes (Kapoor et al., 2011). In particular, the shift in the vibration of CH_2_ groups toward higher wavenumbers was previously linked to an increase in membrane fluidity (Laroche et al., 2005; Alvarez-Ordonez et al., 2010; Passot et al., 2015). Gr3 spectra displayed spectral contributions similar to those of germinated spores in the lipid region. We can draw a parallel between these three groups and the three spore subpopulations identified after HP treatment at 20°C, namely, inactivated, germinated, and sensitized to nisin. Although there are two spore subpopulations after HP treatment at 50°C (inactivated and germinated spores), the spores only form one group with respect to their spectral signature in the lipid region, which is similar to the germinated spores group. In the same way, Gr3 spores include germinated spores and spores inactivated by HP treatment at 20°C. Gr1 and Gr2 spores could thus display the signature of transitory changes arising in the inner or outer membrane of spores after HP at 20°C. The link between such changes occurring in spore membranes and the reversible sensitization of spores to nisin needs to be further investigated.

The same groups of spores were shown in the PCA chart obtained for the amide region. The bands corresponding to α-helix (1,650–1,700 cm^–1^) and β-sheet (1,620–1,630 cm^–1^) structures contributed significantly to the spectral variation between HP_20_ spores and the other populations (Georget et al., 2014). Gr1 spores were divided into two subgroups displaying a spectral signature similar to germinated or dormant spores, whereas Gr2 spores presented all the spectral characteristics of germinated spores. Thus, the spectral characteristics of Gr1 and Gr2 spores seem to represent a transient state that could evolve toward the spectral characteristics of germinated spores. The return of spores to a vegetative cell state is a three-step process: activation, germination, and emergence. External agents like specific pH or temperature conditions trigger the activation step. A mechanical stimulus like abrasion can also activate the spores (Keynan et al., 1964). Even though it is still not well known, a change in the conformation of a receptor protein might lead to this reversible phenomenon (Zhang et al., 2009). That way, spores get ready to wake up. It has never been shown that HP can activate bacterial spores. These changes in the lipid and amide regions could be the signature of the reversible change in the conformation of a receptor protein linked to spore activation. Generally, HP acts simply by promoting the unfolded form of proteins, which is less voluminous than the native form. HP between 100 and 500 MPa has been shown to increase the number and the reactivity of sulfhydryl groups by unfolding the protein structure and exposing the interior sulfhydryl groups (Funtenberger et al., 1997; Zhang et al., 2015).

## Conclusion

In conclusion, our main hypothesis is that the inhibition of spores by nisin after HP is due to the modification of the properties of some IM proteins resulting in better binding of nisin to sulfhydryl groups. Further experiments focusing on the identification of the structures involved in this activation could potentially allow a more subtle physiological characterization of these spores.

## Data Availability Statement

The raw data supporting the conclusions of this article will be made available by the authors, without undue reservation, to any qualified researcher.

## Author Contributions

CM, HS, and J-MP-C contributed to the conception and design of the study. CS and FC organized the data acquisition and contributed to the data analysis. CP and LD performed the statistical analysis. CM wrote the first draft of the manuscript. CP and HS wrote sections of the manuscript. All authors contributed to the manuscript revision and read and approved the submitted version.

## Conflict of Interest

The authors declare that the research was conducted in the absence of any commercial or financial relationships that could be construed as a potential conflict of interest.
